# TRPM2 promotes autophagic degradation in vascular smooth muscle cells

**DOI:** 10.1038/s41598-020-77620-y

**Published:** 2020-11-26

**Authors:** Qiannan Zhao, Jingxuan Li, Wing-Hung Ko, Yiu-Wa Kwan, Liwen Jiang, Lei Sun, Xiaoqiang Yao

**Affiliations:** 1 School of Biomedical Sciences, The Chinese University of Hong Kong, Shatin, Hong Kong, SAR People’s Republic of China; 2 Centre for Cell and Developmental Biology, State Key Laboratory of Agrobiotechnology, School of Life Sciences, The Chinese University of Hong Kong, Shatin, Hong Kong People’s Republic of China; 3 Institute of Pediatrics, Guangzhou Women and Children′s Medical Center, Guangzhou Medical University, Guangzhou, People’s Republic of China; 4 Key Lab of Medical Biotechnology and Ministry of Education, Institute of Cardiovascular Research, Southwest Medical University, Luzhou, Sichuan People’s Republic of China

**Keywords:** Molecular biology, Cardiovascular genetics, Cell biology

## Abstract

Transient receptor potential channel M2 (TRPM2) is a Ca^2+^-permeable channel that is activated by reactive oxygen species (ROS). In many cell types, ROS activate TRPM2 to induce excessive Ca^2+^ influx, resulting in Ca^2+^ overload and consequent cell death. Recent studies suggest that TRPM2 may also regulate autophagy in pericytes and cancer cells by acting on the early step of autophagy, i.e. autophagic induction. However, there is no report on the role of TRPM2 in autophagic degradation, which is the late stage of autophagy. In the present study, we found abundant TRPM2 expression in lysosomes/autolysosomes in the primary cultured mouse aortic smooth muscle cells (mASMCs). Nutrient starvation stimulated autophagic flux in mASMCs mainly by promoting autophagic degradation. This starvation-induced autophagic degradation was reduced by TRPM2 knockout. Importantly, starvation-induced lysosomal/autolysosomal acidification and cell death were also substantially reduced by TRPM2 knockout. Taken together, the present study uncovered a novel mechanism that lysosomal TRPM2 facilitates lysosomal acidification to stimulate excessive autolysosome degradation and consequent cell death.

## Introduction

TRPM2 is a Ca^2+^-permeable cation channel activated by H_2_O_2_, adenosine 5′-diphosphoribose (ADP-ribose) and nicotinic acid adenine dinucleotide phosphate^1,2^. The channel is expressed in neurons, vascular smooth muscle cells, vascular endothelial cells and inflammatory cells^1^. Functionally, TRPM2 channels increase the permeability of endothelial barrier^3^, stimulate inflammatory cytokine production in inflammation cells^4,5^, and promote vascular smooth muscle proliferation and migration^6^.

Numerous studies suggest that TRPM2 mediates ROS-induced cell death^7,8^. ROS activate TRPM2 on the plasma membrane to induce excessive Ca^2+^ influx, resulting in Ca^2+^ overload and consequent cell death in neurons, hematopoietic cells and vascular endothelial cells^1^. Apart from its function in the plasma membrane, TRPM2 is also expressed in lysosomes in pancreatic β-cells and dendritic cells, where it mediates lysosomal Ca^2+^ release^9,10^.

Autophagy is a highly conserved process essential for cell survival under stress conditions including starvation, hypoxia and intracellular stress^11^. Under nutrient starvation, autophagy promotes cell survival by breaking down nonessential cellular components for recycling use^12^. There are several major steps in autophagy, which include autophagosome formation or induction, autophagosome fusion with lysosome, and autolysosomal degradation^11,13^. Autolysosomal degradation is the final step of autophagy, during which autolysosomal substrates are degraded by lysosomal acid hydrolases. These hydrolases, including proteases, lipases and many others, have optimal activity at the acidic pH (pH 4.2–5.3) of lysosome^14,15^. The acidic pH of lysosome and autolysosomes is maintained by vacuolar H^+^-ATPases, which pump H^+^ into the lumen of lysosomes and autolysosomes^14,15^. Nutrient starvation activates vacuolar H^+^-ATPase via AMPK and PI_3_K/Akt pathway to stimulate lysosomal/autolysosomal acidification^16,17^.

Autophagy plays important roles in the health and disease of vascular smooth muscle cells. Alterations in autophagy have been documented in vascular smooth muscle cells in response to various stimuli, resulting in modulation of vascular smooth muscle cell functions, including proliferation, migration, matrix secretion, and differentiation^18^. It is believed that basal and adequate level of autophagy has a protective effect on vascular smooth muscle cells. However, excessive autophagy may cause self-digestion and cell death, which occurs in a variety of vascular diseases including atherosclerosis, restenosis and vascular aging^18^.

Several recent studies have examined the role of TRPM2 in autophagy, but yielded conflicting conclusions^19–21^. TRPM2 was reported to promote autophagy in pericytes^19^ and gastric cancer cells^22^, but inhibit autophagy in Hela cells^20,21^. In gastric cancer cells, TRPM2 was reported to promote autophagy via JNK-dependent pathway^22^, whereas in Hela cells TRPM2 was found to act through Ca^2+^-CAMK2-BECN1 signaling to inhibit the induction step of autophagy^20^. However, all these published studies only focused on the role of TRPM2 in modulating early steps of autophagic flux, namely induction and autophagosome formation. None of the above studies has investigated the role of TRPM2 in the context of autolysosomal degradation, lysosomal acidification or smooth muscle cell autophagy.

In the present study, we studied the role of TRPM2 in starvation-induced autophagic flux in the primary cultured mouse aortic smooth muscle cells (mASMCs). Nutrition starvation is a common way to stimulate autophagy^11^ and it also mimics the microenvironment to which vascular smooth muscle cells are exposed in atherosclerotic plaques^23^. Our results demonstrated that TRPM2 promotes starvation-induced autophagic flux via enhancing autophagic degradation and autolysosomal acidification. Furthermore, TRPM2-mediated excessive autophagic degradation resulted in an increased cell death of mASMCs under starvation. These findings uncovered a novel mechanism through which lysosomal TRPM2 facilitates lysosomal acidification to stimulate excessive autolysosome degradation and consequent cell death.

## Methods and materials

### Mouse aortic smooth muscle cells (mASMCs) primary culture

Wild-type (WT)/TRPM2 knockout (KO) mice were a gift from Yasue Mori Group in Kyoto University, Japan. In TRPM2 KO mice, the trpm2 gene was disrupted by deleting the exon that contributes to the putative pore region of the TRPM2. The mice were of C57BL/6 J background^5^. Some reports showed that TRPM2-related phenotypes have gender difference^24^. Therefore, only male mice were used. Male TRPM2 WT and KO mice were sacrificed by 95% CO_2_ inhalation. Thoracic aorta was dissected out and the surrounding adipose and connective tissues were trimmed off. The dissected aorta was transferred to 6-well plate containing 0.1% (w/v) collagenase type II in HBSS (no Ca^2+^, no Mg^2+^), and then was placed into an incubator at 37 °C for 30 min. The adventitial layer of the aorta was removed with forceps. Vessel lumen was cut open, and the endothelial layer was mechanically removed with curved forceps. The leftover smooth muscle layers were cut into 1–2 mm pieces and transferred to T25 cell culture flask with 1 ml DMEM/F12 (Gibco) containing 10% FBS (Gibco) and 1% antibiotic–antimycotic (Gibco). The flask was placed in 37 °C incubator to allow the aortic pieces firmly adhered to the bottom of flask. Half of the medium was replaced every 3 days. After 7 days, mASMCs were found to migrate out from the aortic pieces. The aortic pieces were removed and the cell incubation was continued with change of media once every 3 days until a confluent layer of mASMCs was formed. The identity of vascular smooth muscle cells was confirmed with fluorescence-labeled anti-α-SMA antibody. Only the cells of 4–8 passages were used for subsequent experiments.

### Induction of autophagy by amino acid starvation

mASMCs were treated with serum-free DMEM for 24 h before starvation. The amino acid starvation was carried out in EBSS (amino acid-free) (Gibco), as described elsewhere^25^. The cells were starved for 3, 9, 16 and 24 h, followed by sample collection for Western blots, TUNEL and other assays. Bafilomycin A1 (Selleckchem) at 30 nM was used to inhibit autophagic degradation.

### Western blots

mASMCs were rinsed with ice-cold PBS once and then lysed in RIPA lysis buffer. The protein concentration of the sample was determined by Bio-rad ABS kit. 20 µg of the protein samples were loaded to each lane and separated in 12% SDS-PAGE gel, then blotted to PVDF membrane. The membrane was incubated overnight with anti-LC3B antibody (Novus; 1:1000 dilution) or anti-p62/SQSTM1 (1:1000; abcam, ab91526) for autophagy detection, or with anti-TRPM2 antibody (Abcam, ab11168, 1:1000 dilution) for expression study, or with anti-caspase-3 antibody (Cell Signaling Technology; 1:1000 dilution) for cell apoptosis detection, or with anti-ATP6V0A1 (Proteintech, 1:1000) for detection of vacuolar H^+^-ATPase. Immunodetection was accomplished with horseradish-conjugated secondary antibodies, followed by ECL detection system (Amersham Pharmacia).

### Autophagic flux detection

mASMCs were incubated with mCherry-GFP-LC3B tandem reporter adenovirus for 3 h and the virus-containing medium was replaced with fresh medium. Starvation experiments were carried out 24 h later. Autophagosomes and autolysosomes were observed under Olympus FV1200 confocal microscope system with 60X oil lens. The yellow puncta represented autophagosomes and red only puncta represented autolysosomes.

### Subcellular immunolocalization

Briefly, the mASMCs grown on coverslips were fixed by 4% paraformaldehyde in PBS, permeabilized with 0.4% Triton X-100, following by incubation with primary antibodies in blocking solution at 4 °C overnight. After three subsequent washes at RT with PBS, the cells were then incubated with fluorochrome-conjugated secondary antibody (1:1000 dilution) for 1 h at room temperature in the dark. TRPM2 (Abcam, ab11168, 1:1000 dilution) and Lamp-1 (Abcam; 1:500 dilution) antibodies were used for TRPM2 and lysosome colocalization detection, respectively. The fluorescence images were acquired with Olympus FV1200 confocal microscope. The colocalization of TRPM2 and Lamp-1 was quantified by Pearson’s correlation coefficient (PCC), which was performed readily using FV1200 software.

### Cytosolic Ca^***2***+^ measurement

The mASMCs were seeded on the confocal dish and loaded with 1 μM Fluo-4/AM (Invitrogen, for H_2_O_2_-induced [Ca^2+^] response) or Fura-2/AM (Invitrogen, for basal Ca^2+^ measurement) in HBSS (Gibco) for 30 min at 37 °C in dark. For H_2_O_2_-induced [Ca^2+^] response, 500 µM–3 mM of H_2_O_2_ were added to activate TRPM2, eliciting extracellular Ca^2+^ entry and lysosomal/autolysosomal Ca^2+^ release. The cells were bathed in 2.5 mM Ca^2+^-HBSS or 0 mM Ca^2+^-HBSS with 1 mM EGTA. 2.5 mM HBSS contained in mM: 140 NaCl, 5 KCl, 2.5 CaCl_2_, 0.4 MgSO_4_, 0.5 MgCl_2_, 4 NaHCO_3_, 0.3 NaHPO_4_, 0.4 KH_2_PO_4_, 6 glucose, pH 7.4. 0 mM Ca^2+^-HBSS did not contain Ca^2+^ but 1 mM EGTA. In some experiments, 200 μM glycylphenylalanine 2-naphthylamide (GPN) (Abcam) was also added to induce the osmotic lysis of lysosomes. Fluo-4 was excited at 488 nm and captured at wavelength of 505–530 nm. Data acquisition was performed using an Olympus FV1000 confocal microscope system. The amplitude of Ca^2+^ response was displayed as a ratio of fluorescence (or maximal fluorescence) relative to the intensity before the application of H_2_O_2_ (F_1_/F_0_ or F_max_/F_0_). Fura-2 was excited by dual excitation wavelength at 340 nm and 380 nm, and the emitted light signal was read at 510 nm. The ratio of F340/F380 was calculated and acquired with MetaFluor imaging software (Molecular Devices).

### Lysosomal acidification measurement

LysoSensor™ Green DND-189 (Invitrogen) was used to measure the lysosomal acidification in mASMCs. The cells were incubated with 1 μM LysoSensor probe for 30 min at 37 °C in dark. The loading solution was replaced with fresh medium and fluorescence was detected by Olympus FV1000 confocal microscope system with 60X oil lens. The excitation and emission wavelength of LysoSensor™ Green DND-189 were 443 nm and 505 nm, respectively. Green puncta with stronger fluorescence intensity represented lysosomes with lower pH value.

### TUNEL assay

TUNEL staining in tissue sections was conducted using the In Situ Cell Death Detection Kit (Roche, Switzerland) as described elsewhere^25^. Briefly, mASMCs were fixed with 4% paraformaldehyde (PFA) and permeabilized with 0.1% Triton X-100 in 0.1% sodium citrate. After washing with PBS, the cells were incubated with terminal deoxynucleotidyl transferase dUTP nick end labeling (TUNEL) reaction mixture for 1 h at 37 °C in a humidified atmosphere in dark. Cell nuclei are counterstained with 4,6 diamidino-2-phenylindole (DAPI). Samples were examined under FV1200 confocal microscope at the excitation wavelength of 488 nm and detection wavelength of 515–565 nm (green). TUNEL- and DAPI-stained nuclei were analyzed using Image J software.

### Animal ethics and experimental methods

All animal experiments were approved by Animal Experimentation Ethics Committee, The Chinese University of Hong Kong. The animal license was issued by The Department of Health, The Government of the Hong Kong SAR. All experimental methods used in this study were performed in accordance with the experimental guidelines and regulations of the Chinese University of Hong Kong.

### Data summary and statistics

All data were shown as mean ± standard error of means (SEM). The statistical significance was evaluated with one-way ANOVA followed by Tukey’s multiple comparison test for comparison of multiple groups. Student’s *t*-test was used for comparison of two groups. Normality test were performed, which confirmed that the data were all of normal distribution. Each data point represented the data from one animal unless indicated otherwise in figure legend. Total animal number in different sets of experiments ranged from 3 to 8 pairs (WT vs. TRPM2 KO) as indicated in respective figure legends. *P *value < 0.05 was considered as statistically significant.

## Results

### Knockout of TRPM2 reduced starvation-induced autolysosomal degradation

**Figure 1:**
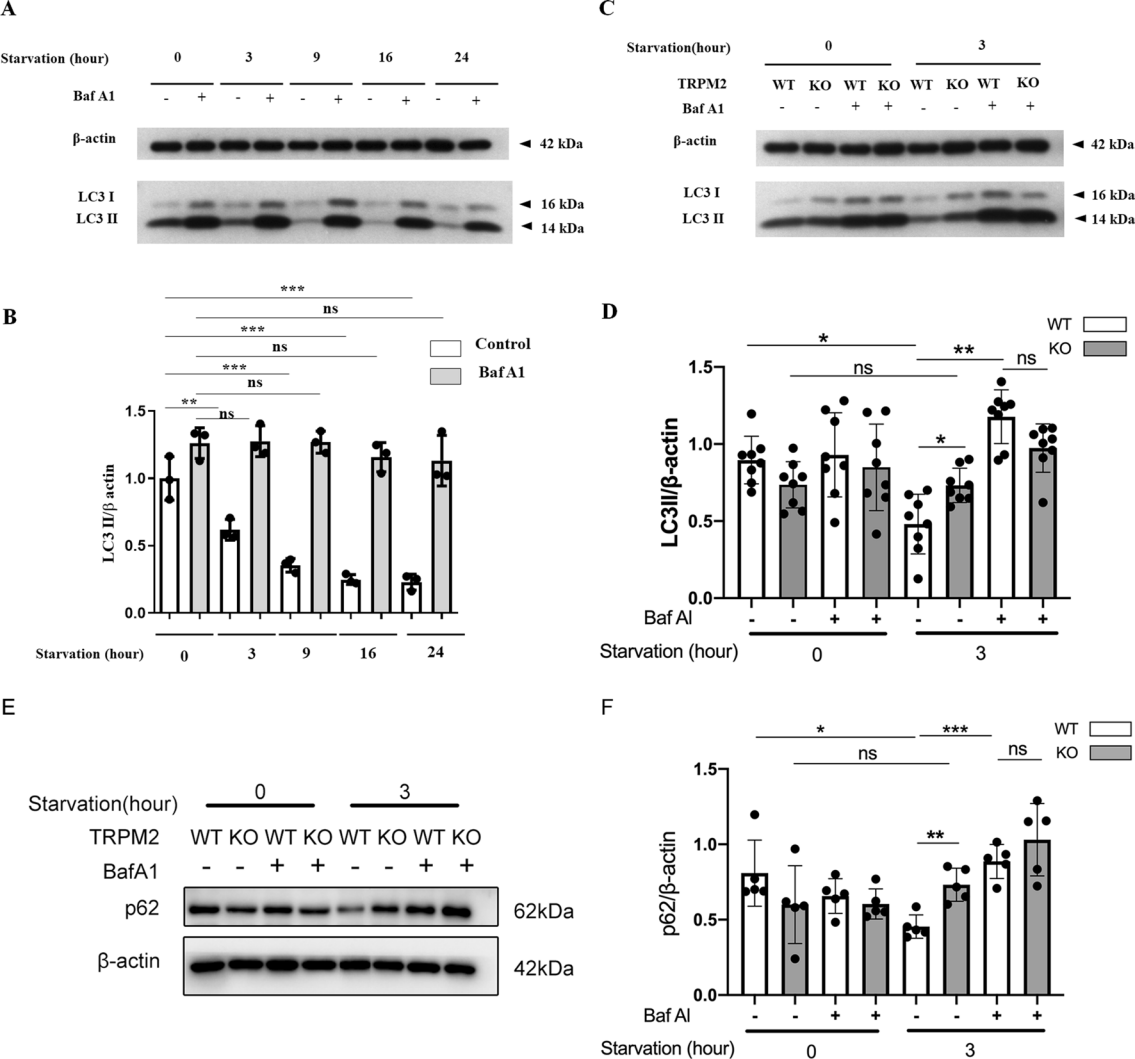
Knockout of TRPM2 reduced starvation-induced LC3 II and P62 degradation. (**A**, **B**) Representative western blot images (**A**) and data summary (**B**) showing starvation (3–24 h)-induced LC3 II change in mASMCs in the presence or absence of bafilomycin A1 (BafA1, 30 nM) treatment. (**C**, **D**) Representative western blot images (**C**) and data summary (**D**) comparing 3 h starvation-induced LC3 II change in mASMCs between WT and TRPM2 KO mice. (**E**, **F**) Representative western blot images (**C**) and data summary (**D**) comparing 3 h starvation-induced p62 change in mASMCs between WT and TRPM2 KO mice. WT stands for wild-type; KO stands for knockout. Values are mean ± SEM (n = 3–8, *P < 0.05, **P < 0.01, ***P < 0.001; ns = not significant). One-way ANOVA was used for statistical test.

Primary cultured mASMCs were subjected to nutrient starvation in HBSS for 3–24 h. Such long periods of starvation from 3–24 h caused progressive decrease in LC3 II level, suggestive of a higher autophagosomal degradation than its formation (Fig. 1A, B). Bafilomycin A1 is an agent that inhibits fusion of autophagosome with lysosome, and it also inhibits vacuolar H^+^-ATPase thus diminishes autolysosomal degradation^12,13^. In the presence of bafilomycin A1, starvation could no longer decrease LC3 II level (Fig. 1A, B), confirming that the decreased LC3 II level under nutrient starvation was the result of an enhanced lysosomal degradation. Autophagic flux, which can be estimated by LC3 II turnover in western blots in the presence and absence of bafilomycin A1, increased progressively as the duration of starvation was prolonged from 0 to 24 h (Fig. 1A, B).

Interestingly, LC3 II reduction in response to 3 h starvation could only be found in mASMCs from WT mice (comparing lane 1 vs. lane 5 in Fig. 1C, D) but not in those from TRPM2 KO mice (comparing lane 2 vs. lane 6 in Fig. 1C, D). Furthermore, at the time point of 3 h starvation, knockout of TRPM2 increased LC3 II level (comparing lane 5 vs. lane 6 in Fig. 1C, D). However, in the presence of bafilomycin, which inhibits autolysosomal degradation, knockout of TRPM2 could no longer increase LC3 II level (comparing lane 7 vs. lane 8 in Fig. 1C, D). p62/SQSTM1 was used as another index for autophagy. Similarly, 3 h starvation reduced the p62 level in mASMCs (comparing lane 1 vs. lane 5 in Fig. 1E, F) but not in those from TRPM2 KO mice (comparing lane 2 vs. lane 6 in Fig. 1E, F). At the time point of 3 h starvation, knockout of TRPM2 increased p62 level (comparing lane 5 vs. lane 6 in Fig. 1E, F). However, in the presence of bafilomycin, knockout of TRPM2 could no longer increase LC3 II level (comparing lane 7 vs. lane 8 in Fig. 1E, F). These data suggest that knockout of TRPM2 reduced autolysosomal degradation under starvation condition, resulting in accumulation of LC3 II and p62. As controls, 3 h starvation or bafilomycin treatment did not alter the expression level of TRPM2 (Fig. S1). However, we did not investigate whether these treatments could change subcellular distribution of TRPM2.

**Figure 2:**
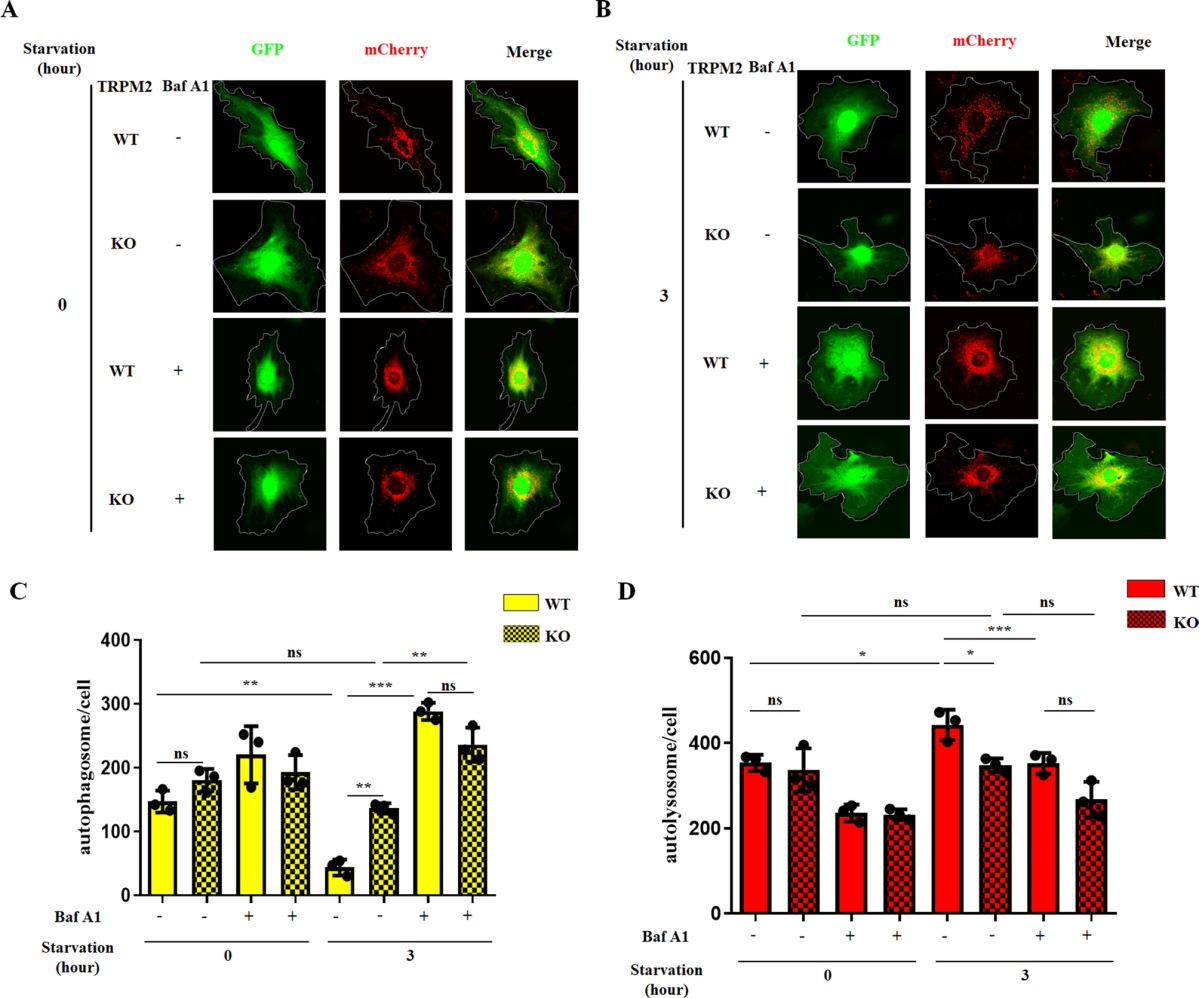
Knockout of TRPM2 reduced starvation-induced change in autophagosomes and autolysosomes. Shown were representative fluorescent images (**A**, **B**) and data summary (**C**, **D**) demonstrating the effect of TRPM2 KO on starvation-induced change in autophagosomes (yellow dots in merged images) and autolysosomes (red alone) in mASMCs. The cells were transfected with adenovirus containing mCherry-GFP-LC3 tandem reporter. 3 h starvation reduced the number of autophagosomes but increased the number of autolysosomes. Knockout of TRPM2 inhibited the starvation-induced reduction of autophagosomes. Values are mean ± SEM (n = 3, *P < 0.05, **P < 0.01, ***P < 0.001; ns = not significant). One-way ANOVA was used for statistical test.

We then transfected mASMCs with the mCherry-GFP-LC3B tandem reporter adenovirus, which could effectively and conveniently monitor autophagic flux^26^. In this assay, autophagosomes were labeled with dual red and green fluorescence, and autolysosomes were labeled with red only^26^. In agreement with the results from western blot analysis (Fig. 1), 3 h nutrient starvation decreased the number of autophagosomes (yellow puncta in the merged pictures of Fig. 2A, B; and lane 1 vs. lane 5 in Fig. 2C) in mASMCs of WT mice. 3 h nutrient starvation also increased the number of autolysosomes (red puncta in the merged pictures of Fig. 2A, B; and lane 1 vs. lane 5 in Fig. 2D) in mASMCs of WT mice. This starvation-induced decrease in autophagosomes was attenuated in mASMCs of TRPM2 KO cells, as reflected by a much higher level of autophagosomes (yellow puncta) in KO cells than WT cells after 3 h starvation (Fig. 2A, B; lane 5 vs. lane 6 in Fig. 2C). As expected, bafilomycin A1 increased the number of autophagosomes (yellow puncta) after 3 h starvation (Fig. 2A, B; and lane 5 vs. lane 7 in Fig. 2C). In the presence of bafilomycin A1, knockout of TRPM2 could no longer increase the number of autophagosomes (comparing lane 7 vs. lane 8 in Fig. 2C).

Collectively, these data suggested that knockout of TRPM2 reduced autolysosomal degradation under starvation condition. In other words, TRPM2 functioned to promote autolysosomal degradation under starvation.

### TRPM2 channels mediated lysosomal Ca^***2***+^ release

**Figure 3:**
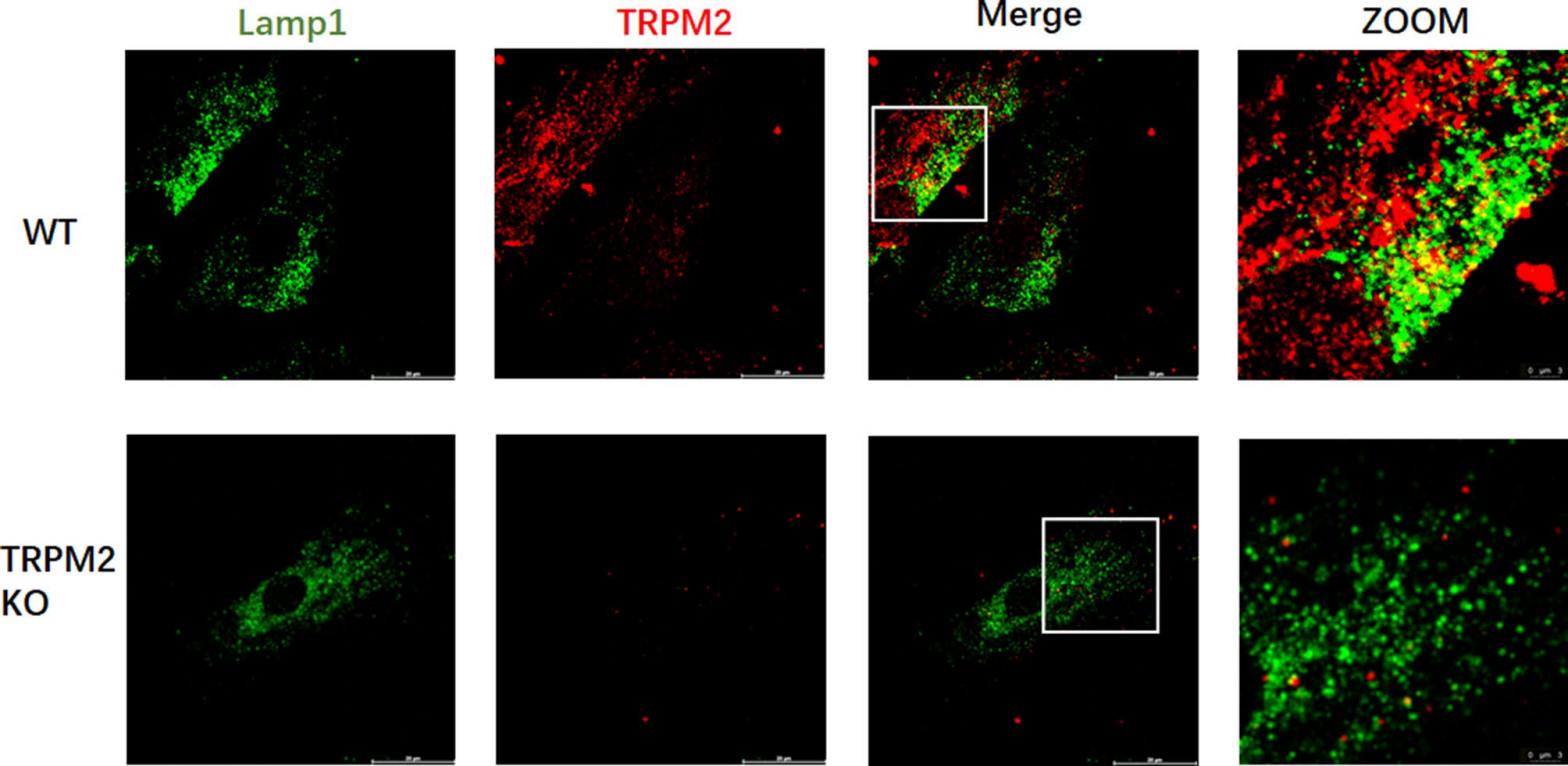
Subcellular co-localization of TRPM2 and lysosome marker Lamp-1 in mASMCs. The cells were immunostained with fluorescence-labeled anti-TRPM2 (Green) and anti-Lamp-1 (red). Red fluorescence represented lysosomes (Lamp-1 positive). TRPM2 expression was found in WT mASMCs, but not in TRPM2 KO mASMCs. n = 3 experiments, 20–30 cells in each experiment.

Subcellular localization of TRPM2 proteins was examined in mASMCs. TRPM2 showed partial co-localization with lysosome marker Lamp-1 (Fig. 3) in mASMCs of WT mice, suggesting lysosomal expression of TRPM2. There was no TRPM2 staining in mASMCs of TRPM2 KO mice, confirming the specificity of anti-TRPM2 antibody (Fig. 3). Quantification of colocalization showed that ~ 28 ± 1% (n = 5) of TRPM2 was co-localized with lysosomal marker LAMP1 in mASMCs of WT mice. Pearson correlation coefficient was 0.52 ± 0.04 (n = 5).

**Figure 4:**
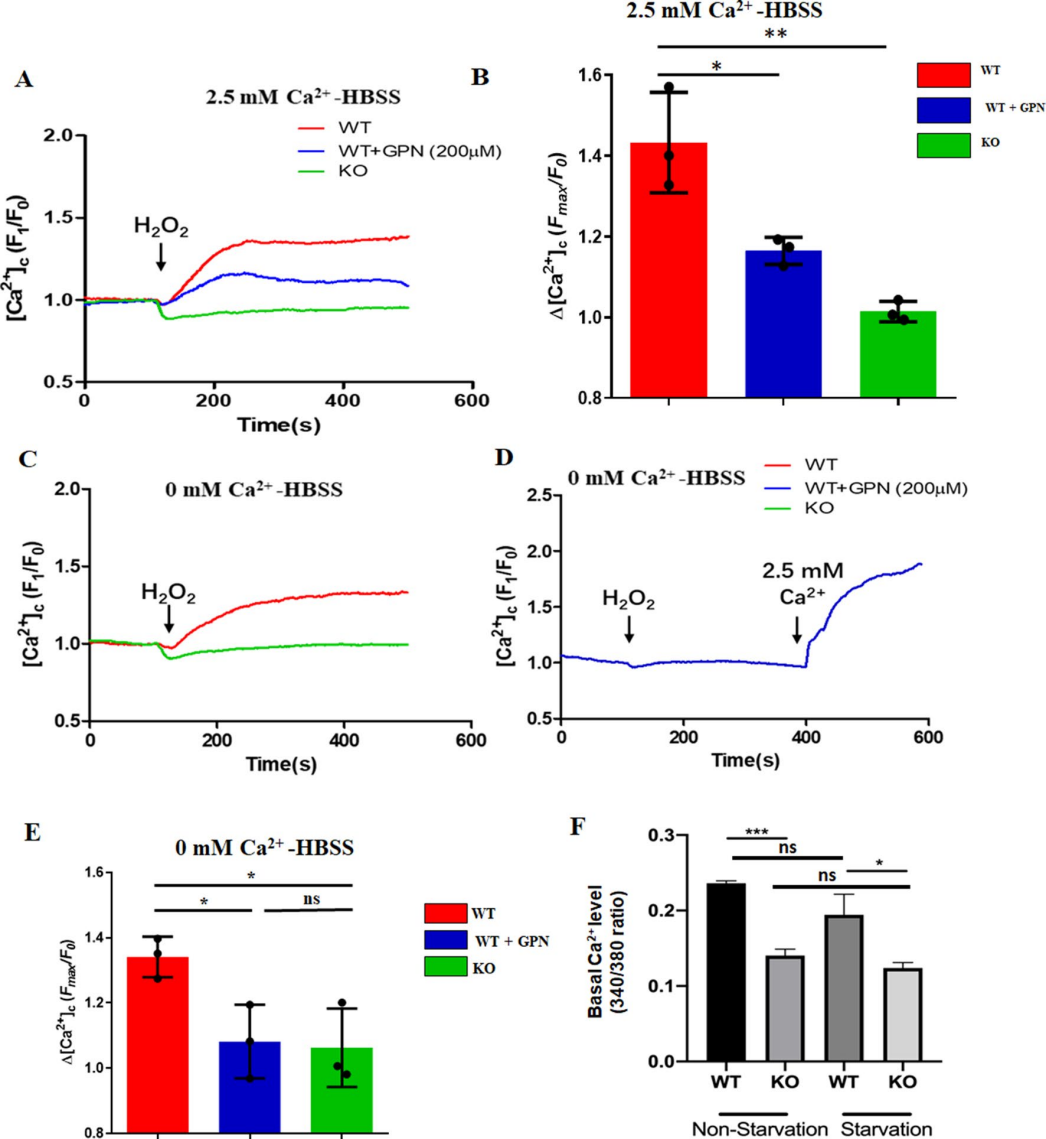
H_2_O_2_ induced cytosolic Ca^2+^ rise by stimulating lysosomal TRPM2. (**A**, **B**). Representative traces (**A**) and data summary (**B**) showing the cytosolic [Ca^2+^] changes in response to 3 mM H_2_O_2_ in mASMCs bathed in 2.5 mM Ca^2+^-HBSS. The H_2_O_2_-induced [Ca^2+^] rise in mASMCs was reduced by lysosome disruption (200 µM GPN) and abolished by TRPM2 KO. (**C**–**E**) Representative traces (**C**, **D**) and data summary (**E**) showing the cytosolic [Ca^2+^] changes in response to 3 mM H_2_O_2_ in mASMCs bathed in 0 mM Ca^2+^-HBSS. The H_2_O_2_-induced [Ca^2+^] rise in mASMCs was abolished either by lysosome disruption (200 µM GPN) or by TRPM2 KO. (**F**) Comparison of basal cytosolic Ca^2+^ level in mASMCs from WT and TRPM2 KO mice under 3 h starvation and non-starvation conditions. Values are mean ± SEM (n = 3 including 8–10 cells in each experiment, *P < 0.05, **P < 0.01, ***P < 0.001, ns = not significant). One-way ANOVA was used for statistical test.

We studied whether TRPM2 could mediate lysosomal Ca^2+^ release. mASMCs were loaded with a Ca^2+^-sensitive fluorescence dye Fluo-4/AM to monitor changes in cytosolic Ca^2+^. The cells were bathed in normal physiological saline HBSS. Challenge of the cells with 3 mM H_2_O_2_ induced a cytosolic [Ca^2+^] rise in mASMCs of WT mice but not TRPM2 KO mice (Fig. 4A, B), confirming that the cytosolic [Ca^2+^] rise in response to H_2_O_2_ was mediated by TRPM2. Importantly, after treating the cells with 200 μM glycyl phenylalanine 2-naphthylamide (GPN) to disrupt lysosomes, H_2_O_2_-induced cytosolic [Ca^2+^] rise in WT mASMCs was markedly reduced (Fig. 4A, B), suggesting that this [Ca^2+^] rise was partly from lysosomal Ca^2+^ release and partly from extracellular Ca^2+^ entry. We also tried lower concentration of H_2_O_2_ and found that H_2_O_2_ at 500 µM could also induce cytosolic [Ca^2+^] rise but at a slower time course and not always reproducible (Fig. S2).

Next, 3 mM H_2_O_2_ was applied to mASMCs bathed in a Ca^2+^-free HBSS containing 1 mM EGTA. Herein, 3 mM H_2_O_2_ could only induce cytosolic [Ca^2+^] rise in mASMCs from WT but not TRPM2 KO mice (Fig. 4C, E). Furthermore, after disruption of lysosomes with GPN, H_2_O_2_ could no longer induced the cytosolic [Ca^2+^] rise in the cells bathed in the Ca^2+^-free HBSS (Fig. 4D, E).

Together, these results demonstrated that TRPM2 was expressed in lysosomes, where it mediated lysosomal Ca^2+^ release in response to H_2_O_2_ challenge.

We also measured the basal level of cytosolic [Ca^2+^] in mASMCs bathed in HBSS. The basal cytosolic [Ca^2+^] was found to be lower in TRPM2 KO cells than in WT cells under both starvation and non-starvation conditions (Fig. 4F), suggesting that TRPM2 contributed to the regulation and maintenance of basal cytosolic [Ca^2+^] level. This was expected because basal activity of TRPM2 should contribute to lysosomal/autolysosomal Ca^2+^ release and extracellular Ca^2+^ entry. However, amino acid starvation for 3 h per se did not significantly alter the basal cytosolic [Ca^2+^] level (Fig. 4F).

### Knockout of TRPM2 attenuated starvation-induced lysosomal/autolysosomal acidification

**Figure 5:**
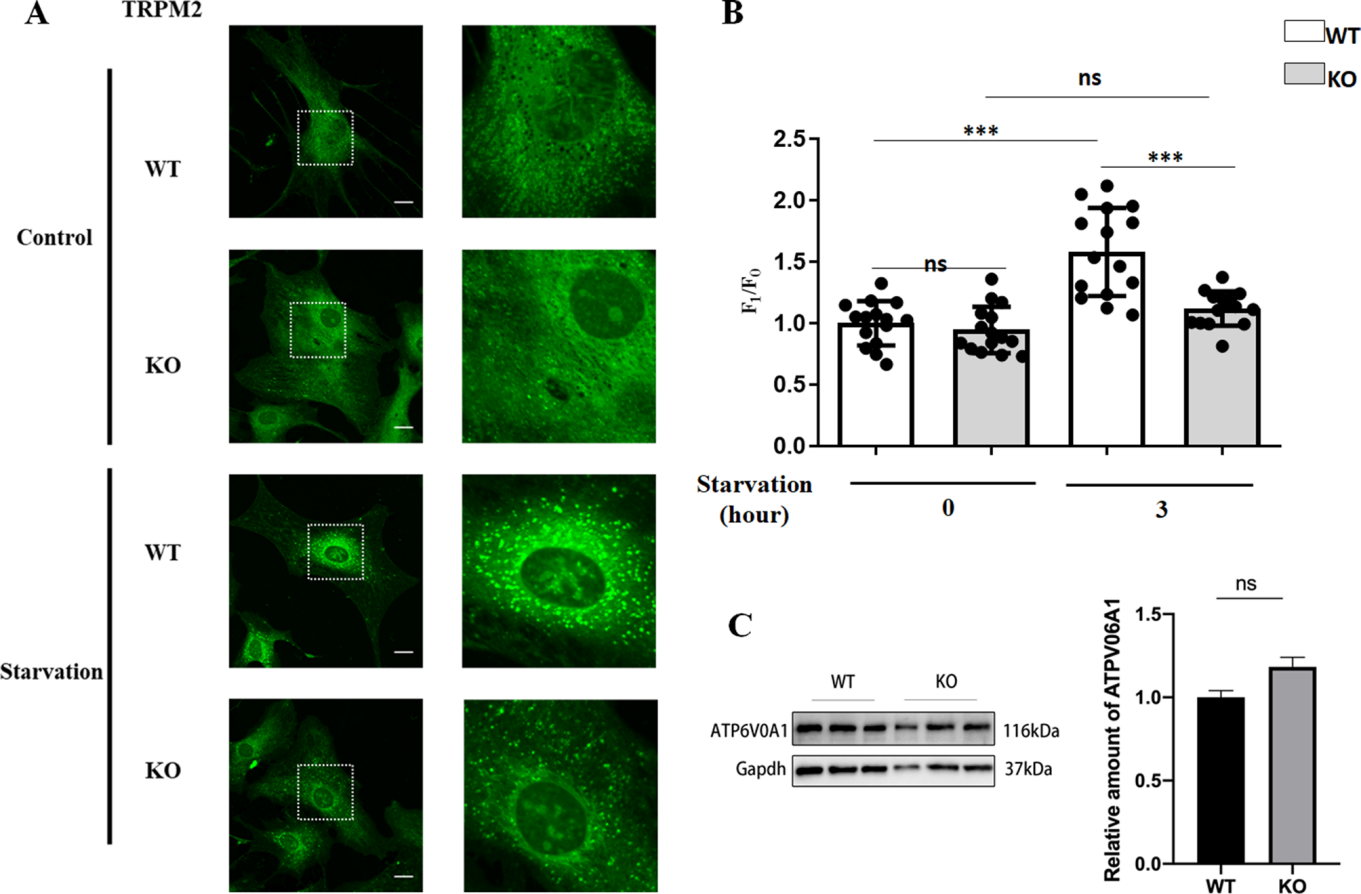
Knockout of TRPM2 attenuated starvation-induced lysosomal/autolysosomal acidification. Shown were representative fluorescent images (**A**) and data summary (**B**) illustrating starvation (3 h)-induced lysosomal/autolysosomal acidification in mASMCs from WT and TRPM2 KO mice. Green puncta represented lysosomes/autolysosomes in cells. Stronger fluorescence intensity indicated lower pH value. Knockout of TRPM2 reduced the starvation-induced lysosomal/autolysosomal acidification. (**C**). Representative western blot images (left) and data summary (right) comparing the expression of ATP6V0A1 in mASMCs from WT and TRPM2 KO mice. Values are mean ± SEM (n = 15 cells from 3 experiments in B, n = 3 experiments in C, ***P < 0.001, ns = not significant). One-way ANOVA was used for statistical test in B. Student t-test was used in C.

LysoSensor Green DND-189 was used to measure lysosomal/autolysosomal acidification in mASMCs. In this method, green puncta represent lysosomes/autolysosomes. Fluorescence intensity of the puncta is positively correlated with the degree of acidification (or lower pH values). Compared to the control group, starvation group showed stronger green fluorescence intensity in lysosomal puncta (Fig. 5A, B), suggesting that starvation promoted lysosomal acidification. Importantly, this starvation-induced acidification of lysosomes/autolysosomes, as indicated by stronger puncta fluorescence, was markedly reduced in TRPM2 KO cells (Fig. 5A, B). These results demonstrated that TRPM2 knockout reduced the lysosomal/autolysosomal acidification under starvation.

Starvation may elevate ROS level^27,28^, which was confirmed by us (Fig. S3). Thus we examined whether ROS could stimulate lysosomal/autolysosomal acidification. However, the results showed that H_2_O_2_ up to 3 mM failed to enhance lysosomal/autolysosomal acidification (Fig. S4). We also compared the expressional level of vacuolar H^+^-ATPase in mASMCs derived from TRPM2 WT and KO mice. The results showed no difference in the expressional level of ATP6V0A1 in mASMCs between TRPM2 WT and KO mice (Fig. 5C). Therefore, the difference in lysosomal/autolysosomal acidification between TRPM2 WT and KO mice was not due to altered expression of vacuolar H^+^-ATPase.

### Knockout of TRPM2 or inhibition of lysosomal degradation reduced the starvation-induced cell death

**Figure 6:**
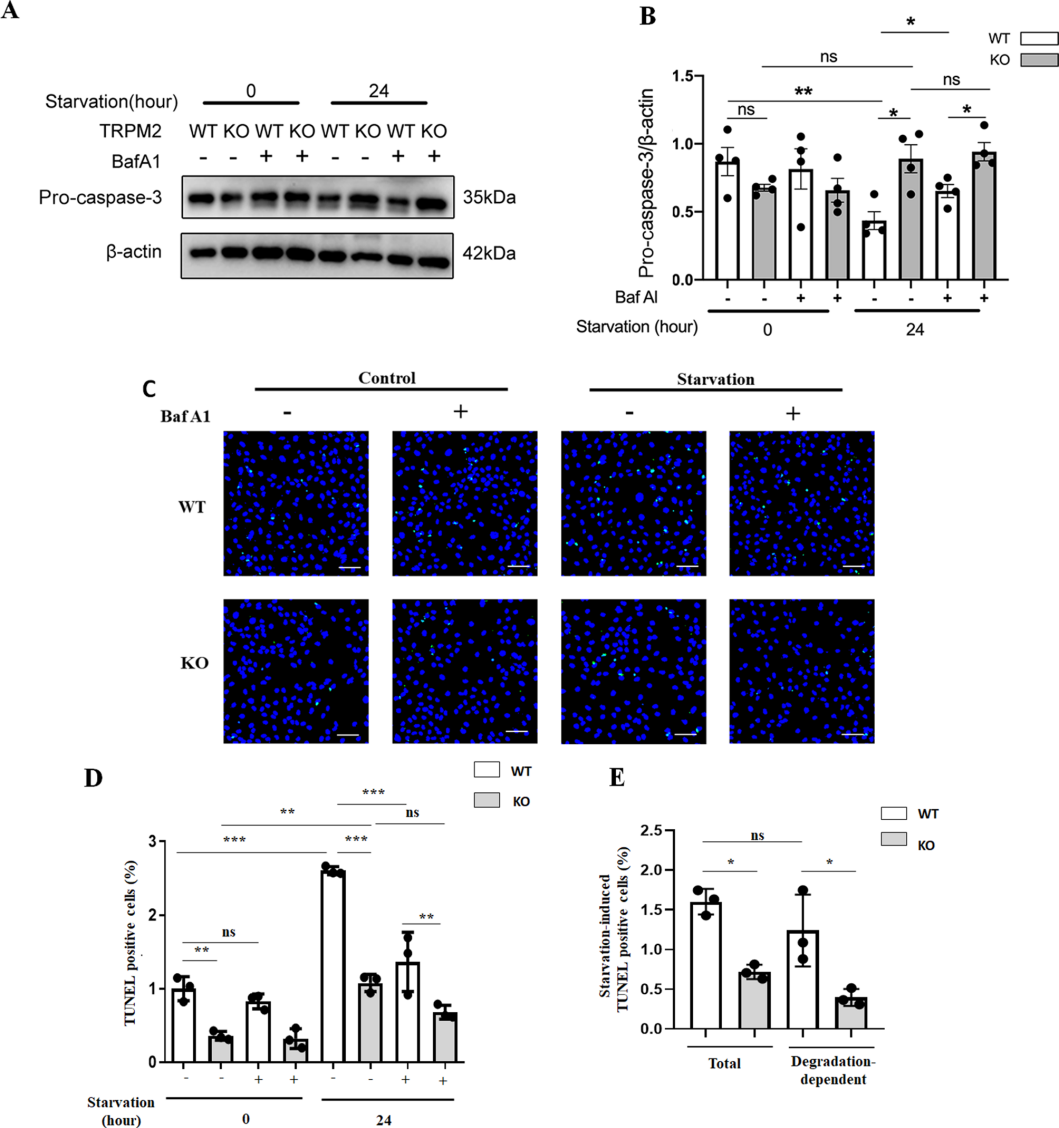
Knockout of TRPM2 or inhibition of lysosomal degradation reduced starvation-induced cell death. (**A**, **B**) Representative western blot images (**A**) and data summary (**B**) showing starvation-induced caspase-3 activation in mASMCs. 24 h starvation decreased the levels of pro-caspase-3 in mASMCs of WT mice, but the effect was absent in mASMCs of TRPM2 KO mice. (**C**–**E**) Representative TUNEL images (**C**) and data summary (**D**, **E**) showing starvation (24 h)-induced cell death of mASMCs. The cells from WT mice showed more cell death compared to the cells from TRPM2 KO mice (**D**, **E**). Inhibition of autophagy by bafilomycin A1 reduced the starvation-induced cell death in both WT and TRPM2 KO cells (**D**). The degradation-related cell death number was also more in cells from WT mice than those from TRPM2 KO mice (**E**). The green signals in C indicated TUNEL-positive apoptotic cells, while blue dots were DAPI counterstain for nuclei. Values are mean ± SEM (n = 3–4, *P < 0.05, **P < 0.01, ***P < 0.001, ns = not significant). One-way ANOVA was used for statistical test.

Next, we determined whether the TRPM2-mediated autophagic degradation were cyto-protective or cytotoxic. Starvation-induced apoptotic cell death in mASMCs was assessed using caspase-3 and TUNEL assays. In caspase-3 assay, 24 h starvation activates caspase-3, as indicated by a decreased level of pro-caspase-3 in western blots (lane 1 vs. lane 5 in Fig. 6A, B). This starvation-induced caspase-3 activation was substantially attenuated by TRPM2 knockout (lane 5 vs. lane 6 in Fig. 6A, B). Similarly, in TUNEL assay, 24 h starvation increased the number of TUNEL-positive apoptotic cells (Fig. 6C, and lane 1 vs. lane 5 in Fig. 6D). This starvation-induced increase in TUNEL-positive apoptotic cells was substantially attenuated by TRPM2 knockout (Fig. 6C, and lane 5 vs. lane 6 in Fig. 6D). Furthermore, bafilomycin A1, which inhibits lysosomal/autolysosomal degradation, also reduced the starvation-induced cell death (lane 5 vs. lane 7 in Fig. 6D). Interestingly, TRPM2 knockout could further reduce the cell death in bafilomycin-treated cells (lane 7 vs. lane 8 in Fig. 6A–D).

We also regrouped the data to illustrate the lysosomal/autolysosomal degradation-dependent cell death under starvation (Fig. 6E). The degradation-dependent component was obtained by subtracting the cell death under bafilomycin A1 treatment from the total cell death. The data clearly showed that TRPM2 substantially contributed to the lysosomal/autolysosomal degradation-dependent cell death (Fig. 6E). Together, these data suggested that, in smooth muscle cells under prolonged starvation, knockout of TRPM2 or inhibition of autophagic degradation have cyto-protective effect. In other words, TRPM2 and autophagic degradation were detrimental to cell survival under prolonged starvation, serving to promote cell death.

## Discussion

The major findings of the present study are as follows: (1) Nutrient starvation stimulated autophagic flux in mASMCs mainly by promoting autophagic degradation. This starvation-induced autophagic degradation was reduced by TRPM2 knockout. (2) At subcellular level, TRPM2-positive immunostaining was found in lysosome. H_2_O_2_ activated lysosomal TRPM2 to mediate Ca^2+^ release. Importantly, starvation-induced lysosomal/autolysosomal acidification was substantially reduced in the mASMCs from TRPM2 KO mice compared with those from WT mice. (3) Starvation-induced cell death of mASMCs was attenuated by TRPM2 knockout. Taken together, the present study uncovered a novel mechanism through which TRPM2 promotes autophagic flux. It is likely that lysosomal TRPM2 facilitates lysosomal acidification to stimulate excessive autolysosome degradation and consequent cell death.

Previous studies have explored the role of TRPM2 in autophagy in several cell types including pericytes, Hela cells and gastric cancer cells, but yielded conflicting results^19–22^. In these studies, TRPM2 was reported to inhibit or promote early steps of autophagic flux, namely induction and/or autophagosome formation^19–22^. However, none of these studies examined the role of TRPM2 in regulating autophagic degradation, which is the final step of autophagic flux. In the present study, we used three independent assays, namely LC3 II and p62 turnovers in western blots and mCherry-GFP-LC3 tandem reporter assay, to determine autophagic flux in mASMCs from WT and TRPM2 KO mice. The results from these assays clearly demonstrated that, under starvation condition, TRPM2 acts to promote autophagic degradation.

We next explored the mechanism of how TRPM2 could promote autophagic degradation. Autophagic degradation occurs in autolysosomes, where targeted substrates are degraded by acid hydrolases^11,13^. These hydrolases have optimal activity at the acidic pH (pH 4.2–5.3). Thus, in general the activity of these hydrolases is positively correlated with the acidity of autolysosomal compartment^14,15^. In the present study, we detected the expression of TRPM2 channels in lysosomes and found the role of these channels in lysosomal Ca^2+^ release in mASMCs. More importantly, we found that starvation-induced acidification of lysosomes/autolysosomes was substantially reduced in mASMCs from TRPM2 KO mice compared with those from WT mice (Fig. 5). Taken together, these data provide strong evidence that lysosomal TRPM2 facilitates lysosomal/autolysosomal acidification, thereby promotes autolysosomal cargo degradation during starvation-induced autophagy. To our knowledge, this is the first report demonstrating a role of TRPM2 in lysosomal acidification.

As for the mechanism of how TRPM2 could facilitate lysosomal/autolysosomal acidification, it is well documented that the acidification of lysosomes and autolysosomes is generated by vacuolar H^+^-ATPase, which pumps H^+^ into the lumen of lysosomes and autolysosomes. Under nutrient starvation, vacuolar H^+^-ATPase is activated via AMPK and PI_3_K/Akt pathway to induce lysosomal/autolysosomal acidification^16,17^. The vacuolar H^+^-ATPase is an electrogenic pump and so the electrogenic gradient generated by the vacuolar H^+^-ATPase must be dissipated by efflux of cations and/or influx of anions to allow sustained proton import^29,30^. Therefore, it is likely that TRPM2-mediated lysosomal/autolysosomal cation (Ca^2+^ and/or Na^2+^) efflux may provide counter current for sustained activity of vacuolar H^+^-ATPase. In other words, TRPM2 has a permissive role to allow vacuolar H^+^-ATPase operating continuously.

It is well documented that basal level of autophagy has protective effect on vascular smooth muscle cells, whereas excessive autophagy may cause self-digestion and cell death of vascular smooth muscle cells, consequently promoting progression of vascular diseases such as atherosclerosis and restenosis^16^. In the present study, we found that, under starvation condition, TRPM2-mediated excessive autophagic degradation may contribute to apoptotic cell death in mASMCs, as indicated by caspase-3 and TUNEL assays. The underlying mechanisms may involve TRPM2-mediated lysosomal/autolysosomal acidification and autolysosomal degradation. We speculate that this mechanism could play a role in atherosclerotic progression. Vascular smooth muscle cells in advanced atherosclerotic lesions have lack of nutrient supply. It is likely that the nutrition deficiency within the atherosclerotic plaques may stimulate the TRPM2-mediated excessive autophagic degradation, leading to the apoptotic death of vascular smooth muscle cells, which could further promote atherosclerotic progression.

Note that previous reports suggested that TRPM2 might also induce apoptotic cell death via other mechanisms independent of lysosomal/autolysosomal degradation^1^. In agreement with this notion, we also found TRPM2 knockout could further reduce the cell death even under the inhibition of vacuolar H^+^-ATPase by bafilomycin (lane 7 vs. lane 8 in Fig. 6A–D). Taken together, the data from us and others suggested that TRPM2 may promote cell death via excessive lysosomal/autolysosomal degradation and also via another mechanism independent of lysosomal/autolysosomal degradation.

In conclusion, the present study identified a novel mechanism that lysosomal TRPM2 induces excessive lysosomal/autolysosomal acidification and degradation to induce apoptotic cell death of mASMCs. We suggest that this mechanism may have important pathophysiological relevance in vascular diseases such as atherosclerosis.

## Supplementary information

Supplementary Figures.
