# Supplementary material for: TRPM2 promotes autophagic degradation in vascular smooth muscle cells
Source: Sci Rep. 2020 Nov 26;10:20719. doi: 10.1038/s41598-020-77620-y (PMC7693237; doi:10.1038/s41598-020-77620-y)
Supplement: Supplementary file 1 — Supplementary Figures. [file 41598_2020_77620_MOESM1_ESM.docx]

**Supplemental Information**

**TRPM2 promotes autophagic degradation in vascular smooth muscle cells**

**Qiannan Zhao^1#^, Jingxuan Li^1#^, Wing-Hung Ko,^1^ Yiu-Wa Kwan^1^, Liwen Jiang^2^, Lei Sun^1,3*^, Xiaoqiang Yao^1,2,4*^**

^1^School of Biomedical Sciences, The Chinese University of Hong Kong, Hong Kong, China

^2^Centre for Cell and Developmental Biology, State Key Laboratory of Agrobiotechnology, School of Life Sciences, The Chinese University of Hong Kong, Hong Kong, China

^3^Heart Center and Institute of Pediatrics, Guangzhou Women and Children’s Medical University, Guangzhou, China

^4^Key lab of Medical Biotechnology and Ministry of Education, Institute of Cardiovascular Research, Southwest Medical University, Luzhou, Sichuan

#: These two authors contribute equally to the work.

*Corresponding authors. Correspondence to [yao2068@cuhk.edu.hk](mailto:yao2068@cuhk.edu.hk) or [lilysun20@gzhmu.edu.cn](mailto:lilysun20@gzhmu.edu.cn).


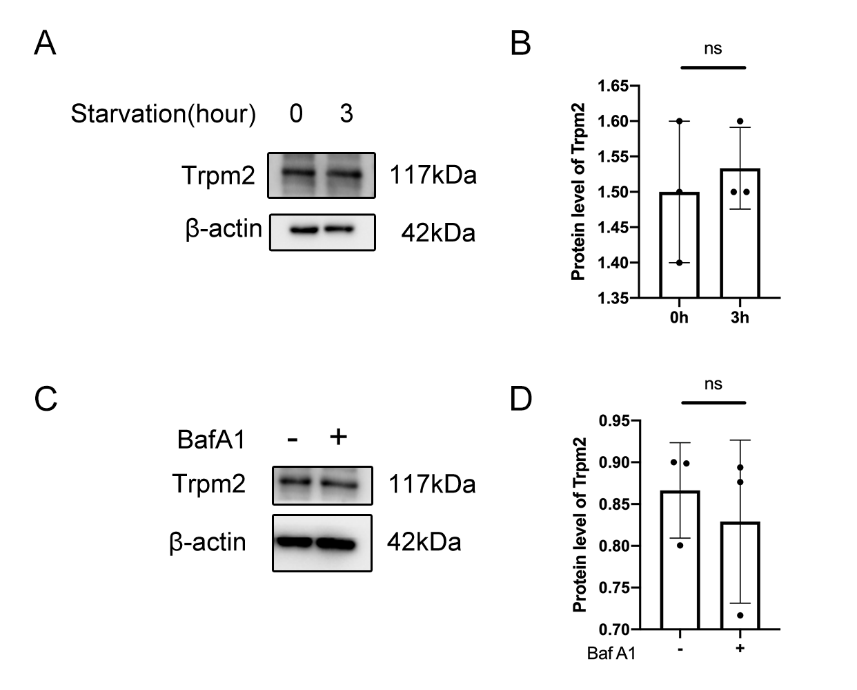


**Fig. S1. Amino acid starvation or bafilomycin treatment for 3 hr did not alter TRPM2 expression in mASMCs.** Shown are the lack of effect for 3 hr starvation (A and B) and bafilomycin (C and D) on TRPM2 protein expression in mASMCs. The amino acid starvation was carried out in EBSS (amino acid-free) (Gibco). Bafilomycin was 30 nM. After treatments, the cell samples were collected for Western blots analysis. Mean ± SEM (n = 3 experiments).

**
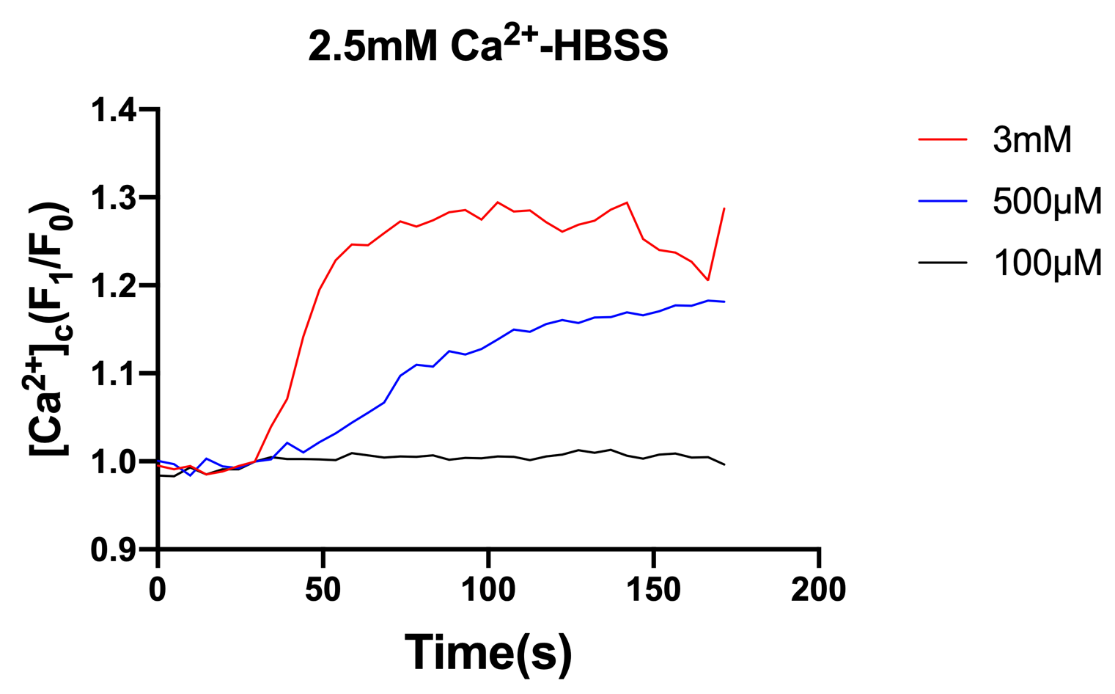
**

**Fig. S2. H_2_O_2_ induced cytosolic Ca^2+^ rise.** Shown are representative traces of cytosolic [Ca^2+^] changes in response to H_2_O_2_ of 100 µM, 500 µM and 3 mM in mASMCs bathed in 2.5 mM Ca^2+^-HBSS. Values are mean ­± SEM (n = 6 including 8-10 cells in each experiment, **P* < 0.05).

**Starvation (hour) (hour)**

**0**

**3**

**WT**


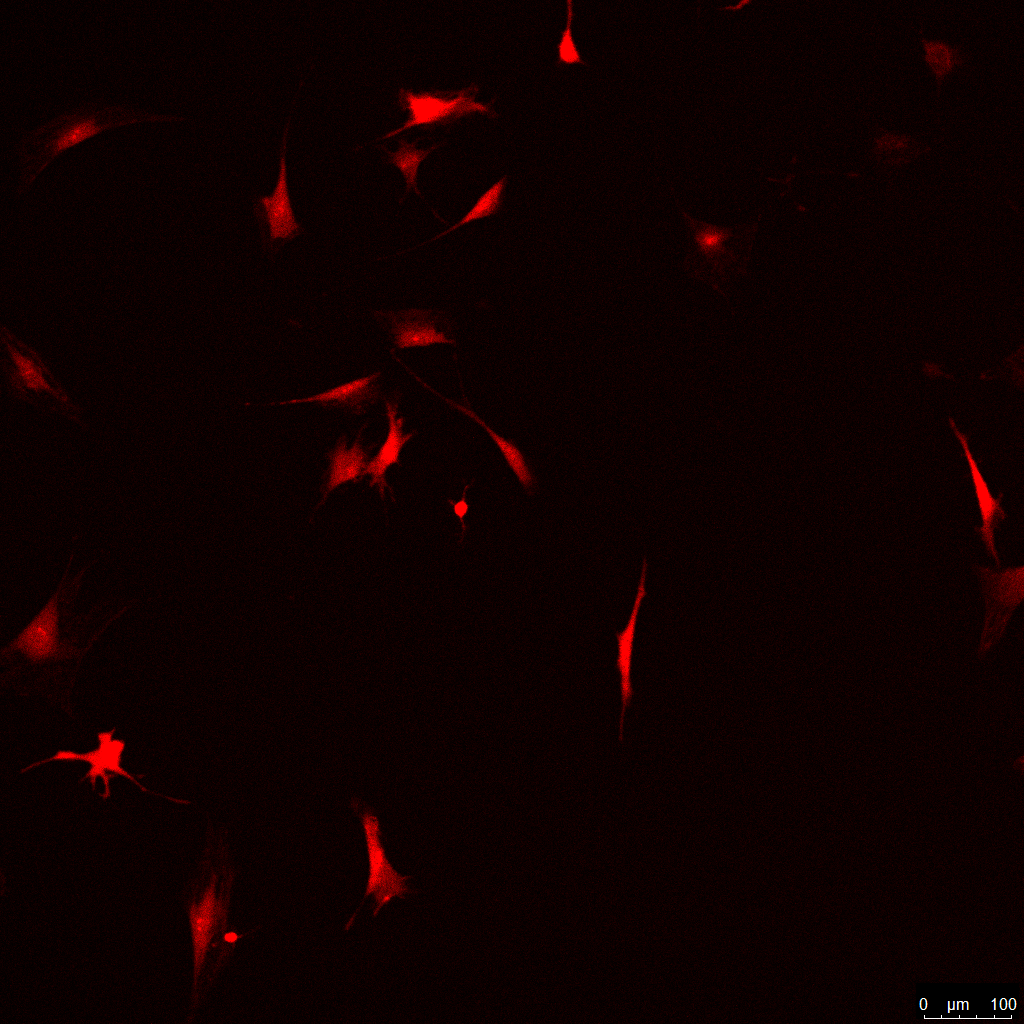

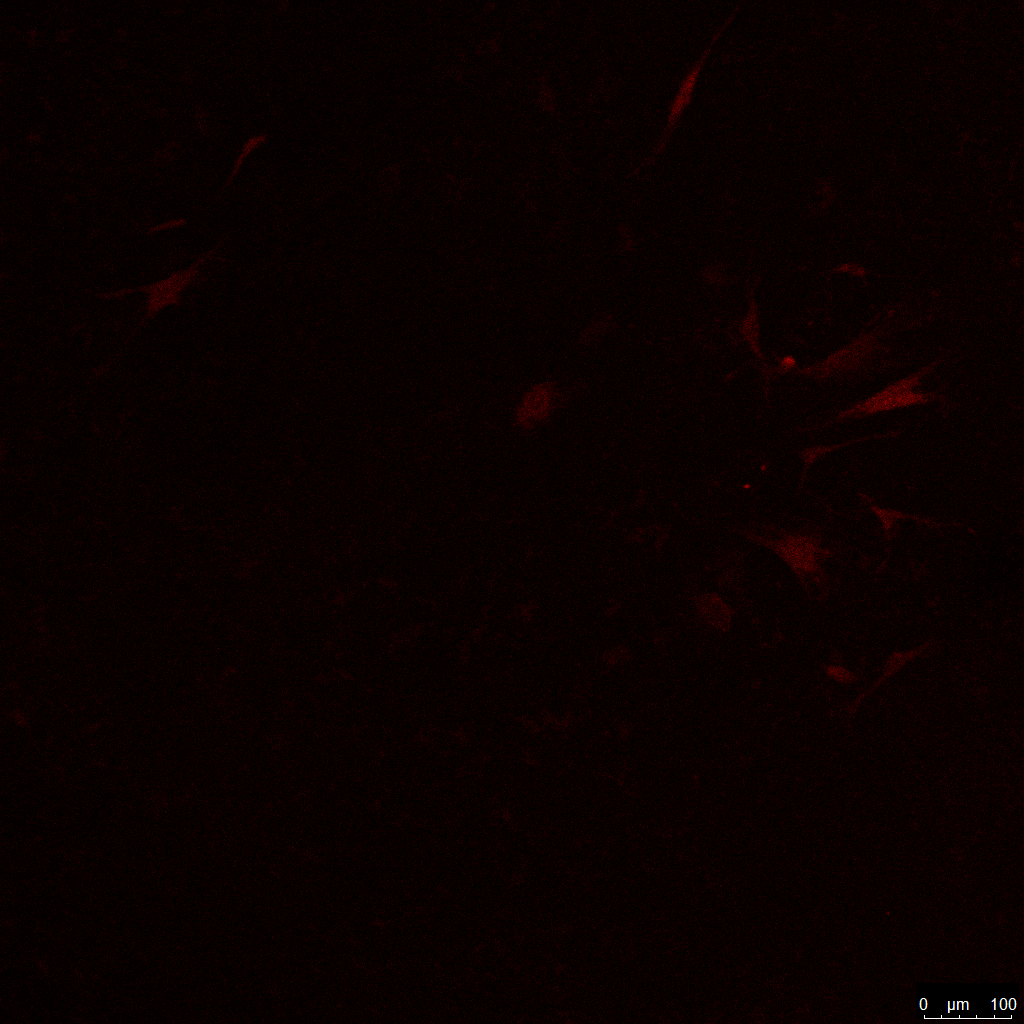


**Fig. S3. Amino acid starvation** **enhanced ROS production in mASMCs.** Amino acid starvation was carried out in EBSS for 3 hr. ROS production in mASMCs was detected by DHE fluorescence. Shown are representative DHE fluorescence images. Experiments were repeated three times.


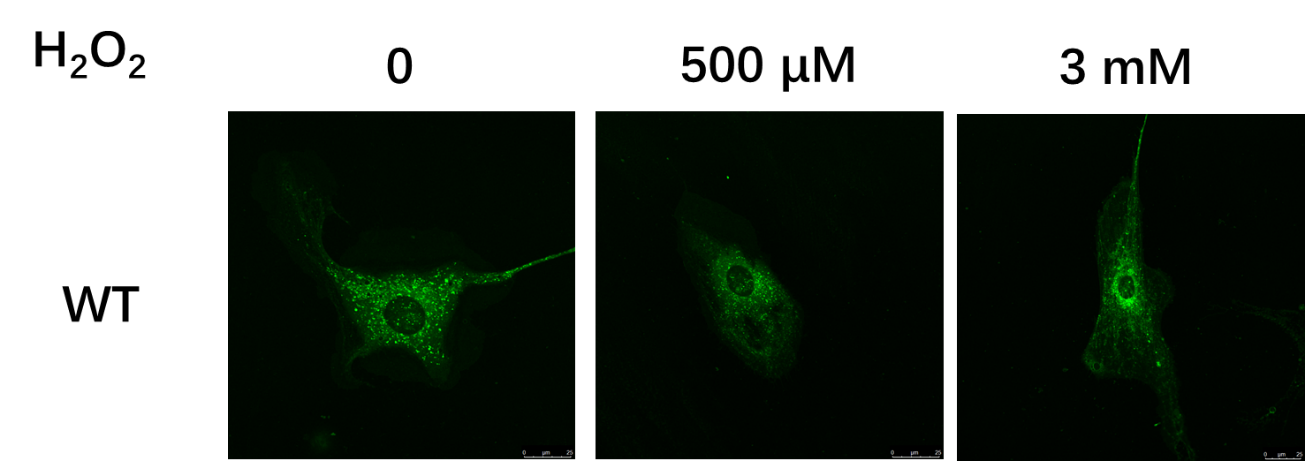


**Fig. S4. H_2_O_2_ up to 3 mM did not cause lysosomal/autolysosomal acidification in mASMCs.** Shown were representative fluorescent images illustrating the lack of stimulating effect of H_2_O_2_ (30 min) on lysosomal/autolysosomal acidification in mASMCs from TRPM2 WT mice. Green puncta represented lysosomes/autolysosomes in cells. Stronger fluorescence intensity was correlated with lower pH value. Experiments were repeated three times.
